# Control of physical properties of carbon nanofibers obtained from coaxial electrospinning of PMMA and PAN with adjustable inner/outer nozzle-ends

**DOI:** 10.1186/s11671-016-1416-7

**Published:** 2016-04-12

**Authors:** Navaporn Kaerkitcha, Surawut Chuangchote, Takashi Sagawa

**Affiliations:** Department of Fundamental Energy Science, Graduate School of Energy Science, Kyoto University, Yoshida-Honmachi, Sakyo-ku, Kyoto, 606-8501 Japan; The Joint Graduate School of Energy and Environment, King Mongkut’s University of Technology Thonburi, 126 Prachauthit Rd., Bangmod, Tungkru, Bangkok 10140 Thailand; Centre of Excellence on Energy Technology and Environment, Science and Technology Postgraduate Education and Research Development Office, Bangkok, Thailand

**Keywords:** Coaxial electrospinning, Core/shell nanofibers, Adjustable nozzle-end

## Abstract

Hollow carbon nanofibers (HCNFs) were prepared by electrospinning method with several coaxial nozzles, in which the level of the inner nozzle-end is adjustable. Core/shell nanofibers were prepared from poly(methyl methacrylate) (PMMA) as a pyrolytic core and polyacrylonitrile (PAN) as a carbon shell with three types of normal (viz*.* inner and outer nozzle-ends are balanced in the same level), inward, and outward coaxial nozzles. The influence of the applied voltage on these three types of coaxial nozzles was studied. Specific surface area, pore size diameter, crystallinity, and degree of graphitization of the hollow and mesoporous structures of carbon nanofibers obtained after carbonization of the as spun PMMA/PAN nanofibers were characterized by BET analyses, X-ray diffraction, and Raman spectroscopy in addition to the conductivity measurements. It was found that specific surface area, crystallinity, and graphitization degree of the HCNFs affect the electrical conductivity of the carbon nanofibers.

## Background

Carbon nanofibers (CNFs) have attracted considerable attention in recent years because they have superior mechanical strength and excellent electronic properties [1, 2] due to their unique structures, which are low grain boundary, 1D structure with high alignment and high surface area to volume ratio. Therefore, because of their unique properties, the CNFs are interesting materials for various applications such as photocatalysts [3, 4], electrodes for supercapacitors [5–7], rechargeable lithium-ion batteries [8–10], photovoltaic cells [11, 12], capacitive deionization process [13], and selective screening [14]. Moreover, their properties can be improved by appropriate selection of precursor material, production process, and treatment. Physical properties of the obtained nanofibers are mostly attributed to their morphology, fiber diameter, and specific surface area.

Some researchers studied the method to increase the specific surface area by fabrication of the nanofibers with a hollow structure [9–16] or increase of porosity [8, 12–16]. Among the methods for preparation of hollow nanofibers, coaxial electrospinning is one of promising techniques. Since it has many advantages such as non-electrospinnable materials can be fabricated, core and shell materials can be flexibly designed to suit with various applications, process instruments or electrospinning conditions also can be adjusted. In addition, it has an ability to form a hollow structure by post spinning removal of core polymer. Hollow carbon nanofibers (HCNFs) produced from bi-component polymer in the coaxial technique represent a new generation of the CNF structures with double the surface area, which benefit to increase the surface area without chemical modification to maintain the chemical stability of the carbon materials [13].

Many studies have been focused on the enhancement of the surface area or the improvement of the morphology of the electrospun nanofibers by improving production process [12], treatment process [6, 17–21], couple of precursor materials [22, 23], precursor solution concentration, type of solvent [24], adjusting the electrospinning conditions including solution flow rate, applied voltage, distance to collector, or characterization techniques for core/shell structure [25–27]. Little attention has been focused on the improvement of coaxial electrospinning nozzle itself. In this study, we used the coaxial nozzles, in which the level of the inner nozzle-end can be adjustable to clarify the effect of the balance level of the inner and the outer nozzle-ends on the physical properties (e.g., morphology, specific surface area, crystallinity, and graphitization degree), which will also affect the electrical properties of the obtained HCNFs.

Many researchers paid effort to clarify the influence of the electrospinning voltage on the properties of the obtained fibers. However, the affection of the applied voltages on the diameter of electrospun nanofibers is a little controversial. Several researchers found that a higher voltage could facilitate an increment of fiber diameters, which may be caused by the faster extraction rate [28, 29]. Some groups, for example, Reneker and Chun [30], reported that the applied voltage has no significant effect to an electrospinning of the nanofibers made of poly(ethylene oxide)s, while some groups, for example, Yuan et al. [31], suggested that an increase of applied voltage results in an increment of electrostatic repulsive force on charged jet. This stretching then causes the smaller diameters of the obtained nanofibers. Not only the fiber diameter but also other properties such as degree of alignment and probability of the formation of beads were affected by the applied voltage. For example, Rouhollah et al. [32] demonstrated that electrospinning jet start to form at low applied voltage of 8.5 kV though electrostatic forces was not strong enough to keep continuous jet. By increasing the applied voltage, the degree of alignment of the nanofibers was also increased. The optimum voltage for electrospinning of PAN nanofibers was between 10–13 kV, while the effects of voltage on the formation of beads on the nanofibers were studied by Deitzel et al. [33]. From their results, along with the increase of the applied voltage, the density of beads was increased. The authors attributed this result to the change of originated jet fluid at the nozzle-end. The pull-outed solution and the supplied solution from the syringe-pump at the nozzle-end was imbalanced resulting in the formation of the beads. Controversially, Zuo et al. [34] studied the effect of applied voltage on the bead formation. They found that with the increase of the voltage from 10 to 26 kV, the average size of the beads in the PHBV fibers were reduced from 14 to 8 μm, respectively. These findings can be concluded that when all related parameters were maintained constant, however, the optimum range of the applied voltage still varies and depends upon internal factors such as the components of electrospinning fluid including various kinds of polymers and solvents.

For the coaxial electrospinning, the effect of applied voltage might align in the same trend. Many research groups demonstrated that when the applied voltage is increased, the diameter of the fibers was decreased. In the meanwhile, the core diameter of electrospun core/shell fibers was also decreased [35, 36]. Viness et al. [37] reported that the diameters of the electrospun fibers were decreased, and by increasing the applied voltage up to some saturated values, then the fiber diameters were increased. In this work, the applied voltage for the three types of nozzle-ends for coaxial electrospinning was optimized in terms of preparation of homogeneous carbon nanofibers with high electrical conductivity.

## Methods

### Preparation of the HCNFs

**Fig. 1:**
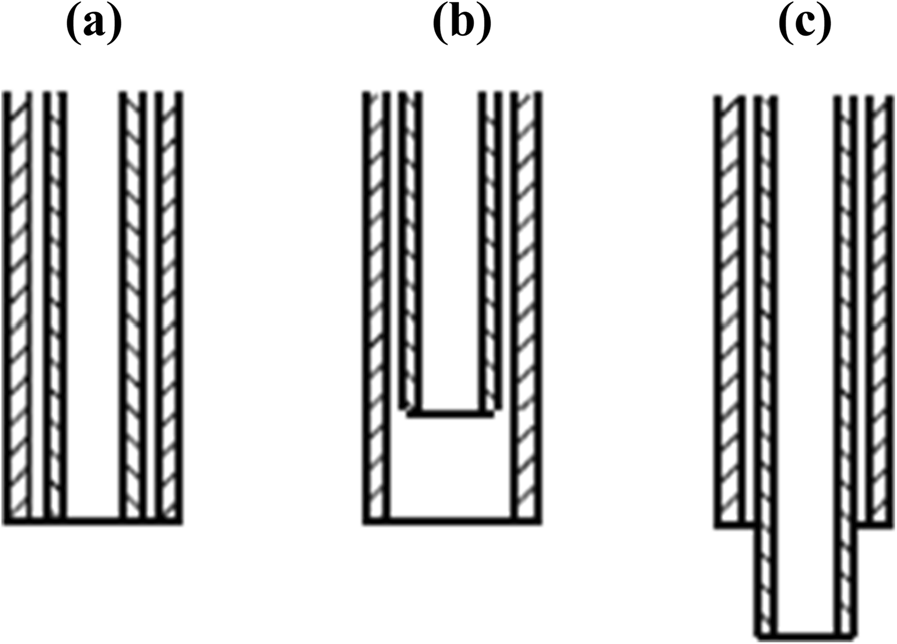
Schematic diagrams of the nozzle-ends. **a** normal coaxial nozzle (viz*.* inner and outer nozzle-ends are balanced in the same level), **b** inward coaxial nozzle, and **c** outward coaxial nozzle

Poly(methyl methacrylate) (PMMA; *M*_w_: 996,000 g mol^−1^, Sigma-Aldrich) and poly(acrylonitrile) (PAN; *M*_w_: 150,000 g mol^−1^, Sigma-Aldrich) were used as thermally degradable core precursor and carbonizing shell precursor, respectively. The polymers were dissolved separately in *N*,*N*-dimethylformamide (DMF; 99.5 %, Wako), where the concentration of PMMA and PAN were 10 and 12 wt%, respectively. Composite nanofibers of PMMA/PAN were prepared by in-house designed coaxial nozzle electrospinning which adjustable inner nozzle-end for 1 mm inward and outward direction compared to the outer nozzle. To study the influence of inner/outer nozzle-end level, the inner nozzle was set to three levels as shown in Fig. 1. The outer and inner diameters of the nozzles are 1.20 and 0.58 mm, respectively. The nozzle-end-to-collector distance was 20 cm, and the flow rates of inner (PMMA) and outer (PAN) precursor solutions were 1.0 and 2.0 mL h^−1^, respectively. The applied voltage was varied from 10 to 25 kV to study its effect on the morphologies of the obtained PMMA/PAN composite nanofibers, as spun PMMA/PAN composite nanofibers were thermally treated for oxidative stabilization for 30 min after increasing temperature to 250 °C at a rate of 5 °C min^−1^ in air, carbonized for 1 h at 800 °C in nitrogen, and finally heated at 1000 °C in nitrogen for another hour to obtain the HCNFs. For comparison, the normal CNFs were also prepared by electrospinning of PAN solution (12 wt%) using single nozzle (20 G, 0.9 mm) with a flow rate of 1.0 mL h^−1^ under the same electrospinning conditions. The stabilization and carbonization process was also carried out under the same conditions as the HCNFs.

### Characterization

The morphologies of the samples were observed by field emission scanning electron microscopy (FESEM; Hitachi SU-6600). High-resolution images were obtained from transmission electron microscopy (TEM; JEOL JEM-2100) operated at 200 kV. The physical adsorption properties were determined by N_2_ adsorption/desorption measurements (BEL Japan, BELSORP 18). The Brunauer-Emmet-Teller (BET) method was used to determine the specific surface area (SSA), the pore volume and the pore size of the HCNFs and CNFs. The crystallinity and the degree of graphitization were investigated by X-ray diffraction analysis (XRD; Rigaku, Smartlab) and Raman spectroscopy (Ocean Optics, QE Pro High Performance Spectrophotometer, *λ* = 785 nm). From C(002) peak of XRD spectra, we can calculate the interlayer spacing (c/2) of the carbon nanofiber by Bragg’s law and the crystallite size along the *c*-axis (*L*_*c*_) by Scherrer’s equation. The graphitization degree of the carbon nanofiber was determined by *I*_d_*/I*_g_ ratio, which is the ratio between the peak intensity of the disordered carbon (1355 cm^−1^) and graphitic carbon (1575 cm^−1^) from Raman spectrum [38], the crystallite size along *a*-axis (*L*_*a*_) then were calculated from *I*_d_*/I*_g_ ratio.

The conductivity measurement was carried out using two-electrode cells. A paste of grinded carbon nanofibers were prepared by mixing 0.1 g of HCNFs and 0.04 g of polyethylene glycol (PEG; *M*_w_: 7300∼9300, Wako) into 0.6 mL of ethanol/water. The HCNF paste was spread on an ITO-coated glass substrate (Geomatech, 10 Ω/sq, thickness 1.1 mm) by the doctor-blade technique using adhesive tape masking to control the thickness. The concise layer thickness was measured by cross-sectional SEM images. An aluminium electrode (thickness 100 nm) was thermally evaporated on the samples, and then the current density-voltage (J-V) characteristic (Bunkoukeiki, IV-2401) is measured to obtain the conductance (G, Siemens) values. Then the conductance value was used to calculate the electrical conductivity of the samples.

## Results and Discussion

To confirm the effect of applied voltage on the PMMA/PAN composite nanofibers obtained from three different nozzle-end configurations. The applied voltage was varied from 10 to 25 kV while the other factors including the collecting distance and solution flow rates were kept constant. The morphologies of obtained as spun composite nanofibers were shown in Table 1.

**Table 1:**
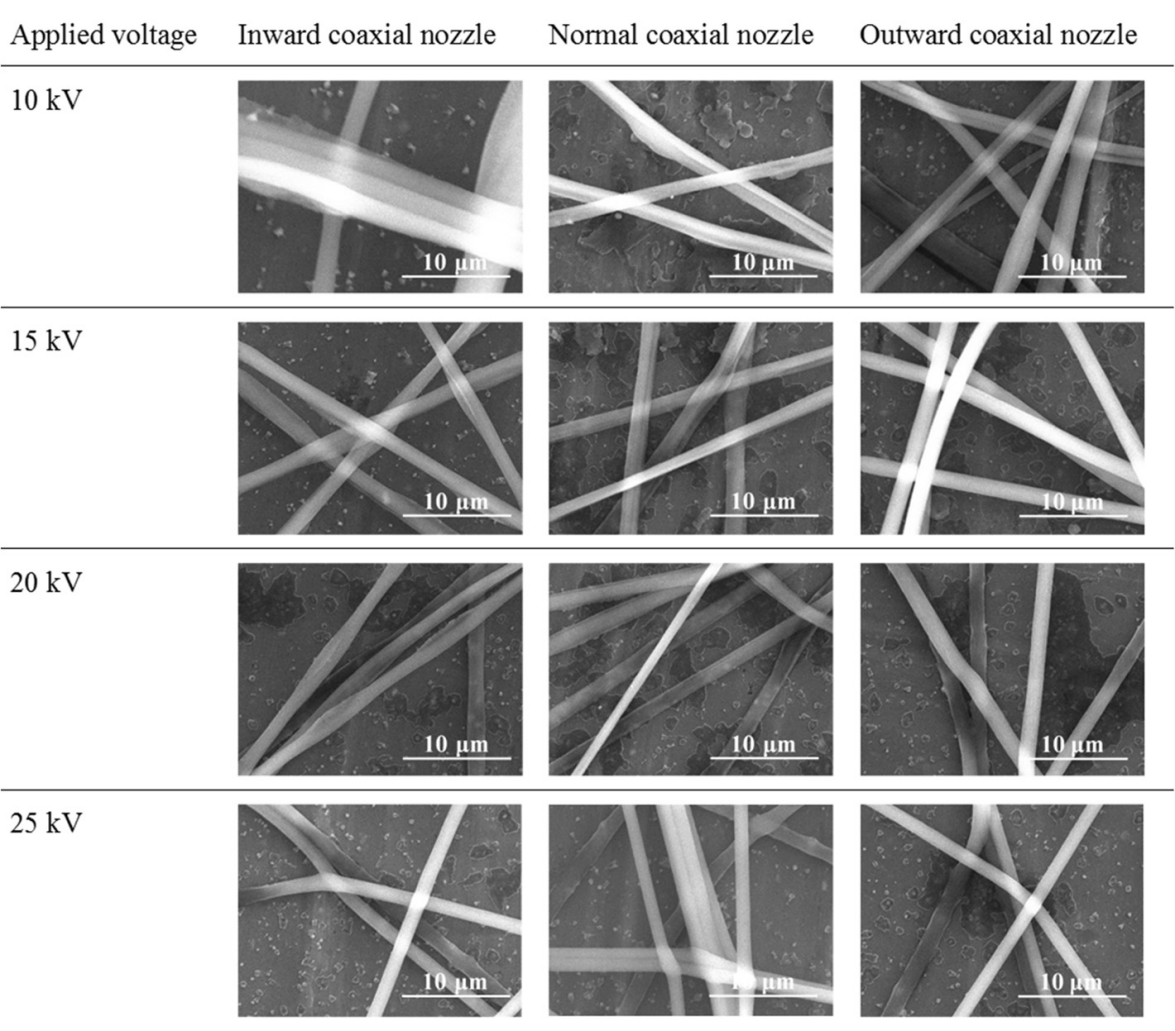
SEM images of the as spun nanofibers as a function of applied voltage

As indicated in SEM images, the effect of voltage shows the same trend for all three configurations of the nozzle. The good morphologies of PMMA/PAN nanofibers (smooth and uniform in the distribution of the sizes) were obtained from the coaxial nozzle electrospinning at 15 kV. At a lower applied voltage of 10 kV, the injection was occurred after an electrospinning solution was agglomerated at the end of the nozzle, retarded for a while until the electrostatic force was strong enough to inject the solution jet. The initial agglomerated shape may cause some difficulties for the solution jet to form the continuous compound of Taylor’s cone at the end of the nozzle. Therefore, the obtained nanofibers show a non-uniform beaded fiber morphologies and some nanofibers were fused together as a bundle of fibers. For the coaxial electrospinning at higher voltage of 15–25 kV, composite nanofibers with no beads and more uniform morphologies were obtained. However, when the applied voltage was increased greater than 20 kV, the extraction rate of the precursor solution was too high and the solution was collected and accumulated at the collector before the evaporation of the solvent. Therefore, wet nanofibers were fused onto the aluminium foil collector or fused together resulting in the bundle morphologies. From the obtained results, we used the applied voltage at 15 kV for further experiment.

**Fig. 2:**
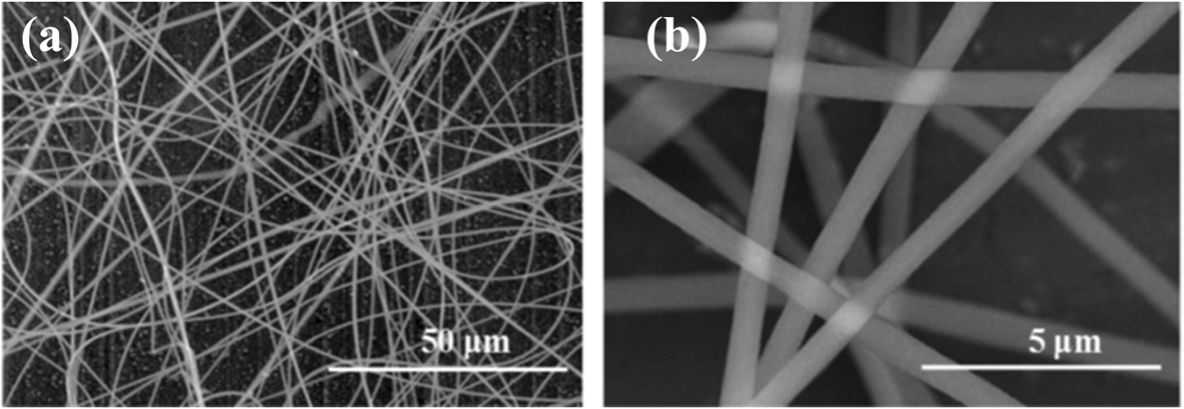
SEM images of the as spun PAN nanofibers from single nozzle electrospinning. **a** at low magnification and **b** at high magnification

For comparison, the morphologies of as spun PAN nanofibers from single nozzle electrospinning were also observed by SEM as shown in Fig. 2. The PMMA/PAN nanofibers from all three types of coaxial nozzle-configurations (at voltage of 15 kV) (Table 1) and PAN nanofibers (Fig. 2a) exhibit continuous fibrous morphology with diameters ∼1.16 and ∼0.80 μm, respectively. However, we can observe some wrapped morphology on the PMMA/PAN nanofibers, which was generated by the instability of the jet fluid during electrospinning [6] because of the difference in solubility and conductivity of the inner and outer polymer solution. Whereas for the PAN nanofibers, uniform morphology and relatively smoother surface was observed as shown in Fig. 2b.

**Fig. 3:**
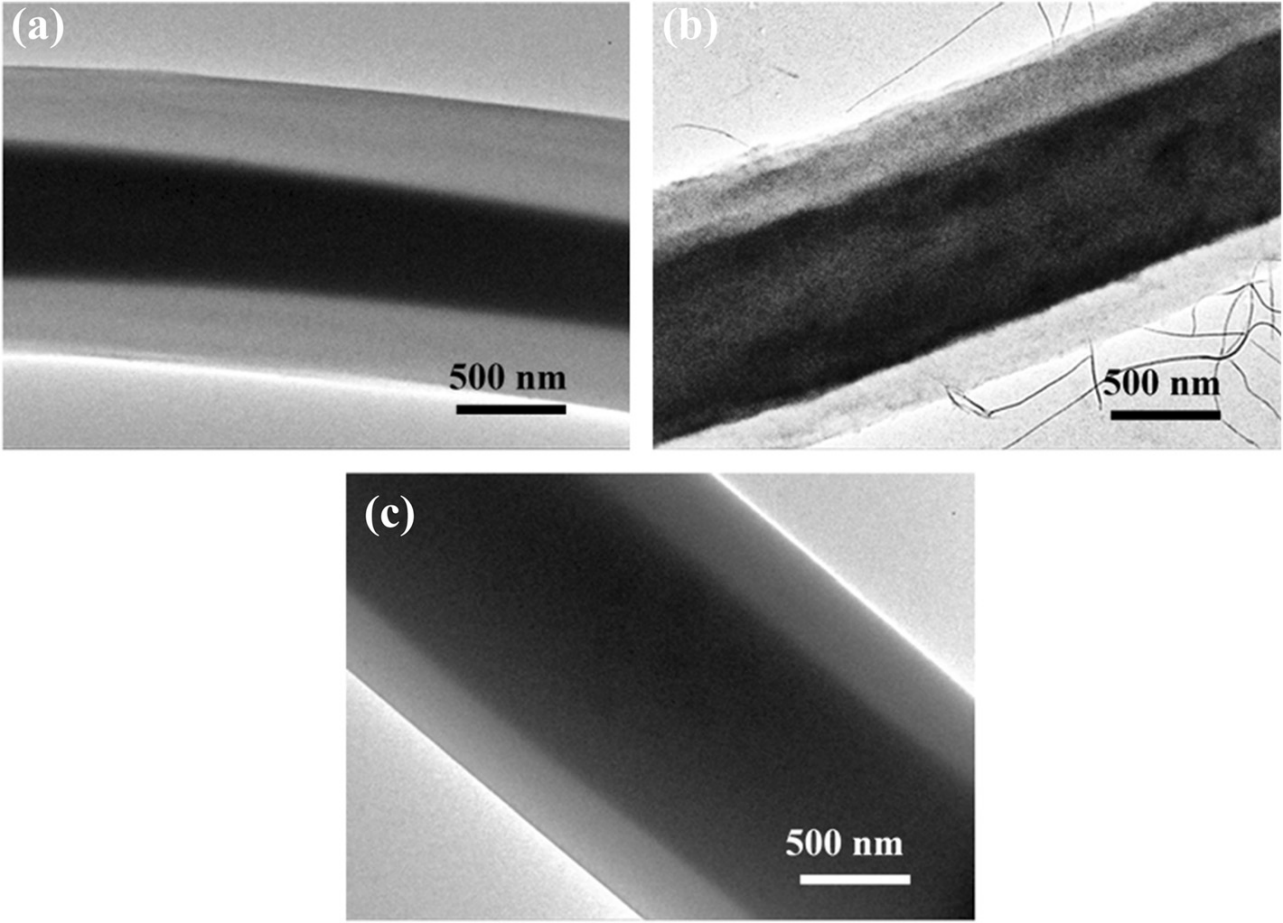
TEM images of the as spun PMMA/PAN nanofibers. **a** normal coaxial nozzle, **b** inward coaxial nozzle, and **c** outward coaxial nozzle

The TEM images of the as spun PMMA/PAN composite nanofibers obtained from coaxial electrospinning with different inner nozzle-end levels were shown in Fig. 3. All coaxial nozzle electrospinning samples exhibit the core-shell morphology, which the core portions were uniformed along the center of the nanofibers. The overall diameter and core diameter of the PMMA/PAN nanofibers are summarized in Table 2. From the table, the PMMA/PAN nanofibers produced from outward coaxial nozzle reveal the largest nanofiber diameter and smallest wall thickness compared to normal and inward coaxial nozzle systems. This may be due to the inner nozzle-end which was set to the closer distance to the aluminium collector, generating more electrostatic force that pulled the inner fluid out of the nozzle. At the fixed electrospinning flow rate of core and shell solutions, the pulled out rate of both precursor solutions in the outward coaxial nozzle system might be much higher than those in the cases of normal and inward coaxial nozzle system, leading to a larger diameter of the nanofibers. In addition, the inner fluid was pulled out at a faster rate than the outer fluid, resulting in the stronger shear force between inner and outer fluid during coaxial electrospinning. Therefore, the PMMA/PAN nanofibers obtained from the outward coaxial nozzle system reveal much smaller wall thickness than the other systems and thus can improve their specific surface area.

**Table 2:** Diameters and wall thickness of the HCNFs before and after carbonization process

Nozzle-end level	Before carbonization			After carbonization			Shrinkage (%)
	Overall diameter (μm)	Core diameter (μm)	Wall thickness (μm)	Overall diameter (μm)	Core diameter (μm)	Wall thickness (μm)	
Inward	1.42	0.82	0.26	0.72	0.31	0.22	49.30
Normal	1.38	0.57	0.39	0.53	0.29	0.11	61.59
Outward	1.88	1.04	0.36	0.47	0.27	0.14	75.03

After electrospinning, the as spun nanofibers were stabilized and carbonized to obtain the carbon nanofibers. In this process, the as spun nanofibers were stabilized in oxygen atmosphere for a greater stability to sustain at higher temperature [17]. When increasing the temperature, the PAN crystal structure was almost completely destroyed and a thermally stable ladder polymeric structure was formed. The hollow structure in the HCNFs after thermal treatment was generated by thermal stability difference between the two precursor solutions. At high temperature, the PMMA core portion was totally decomposed to gaseous products without remaining residual material [13]. The gaseous products were leaked out of the nanofibers which also leads to an increment of the microporous structure on the surface of the HCNFs [8].

**Fig. 4:**
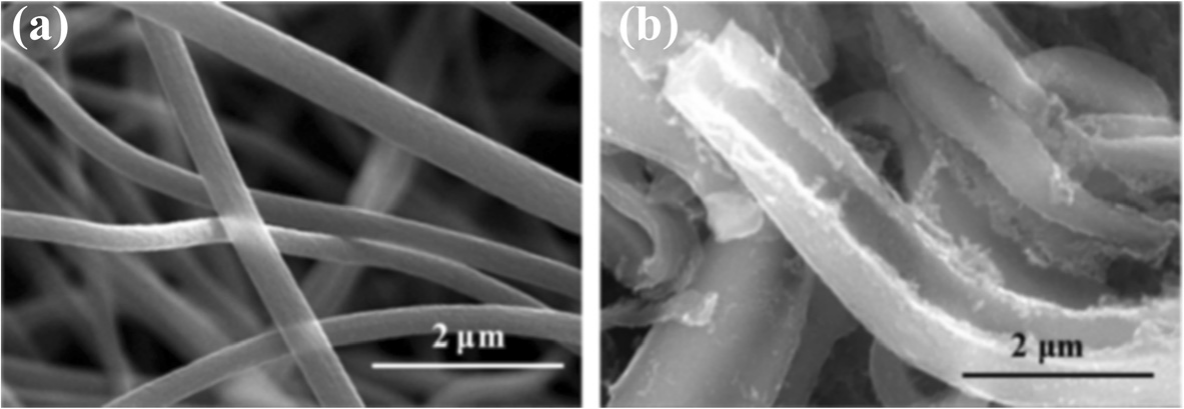
SEM images of the carbon nanofibers after carbonization. **a** CNFs from single nozzle and **b** HCNFs from normal coaxial electrospinning

The SEM images of the surface of the CNFs and the HCNFs after the stabilization and carbonization process show some rough surface as shown in Fig. 4. For the HCNFs, the hollow morphology could be observed in some nanofibers (as shown in Fig. 4b). This may occur if the core portion of the nanofiber was not at the center, but on the edge of the PMMA/PAN nanofibers during electrospinning.

**Fig. 5:**
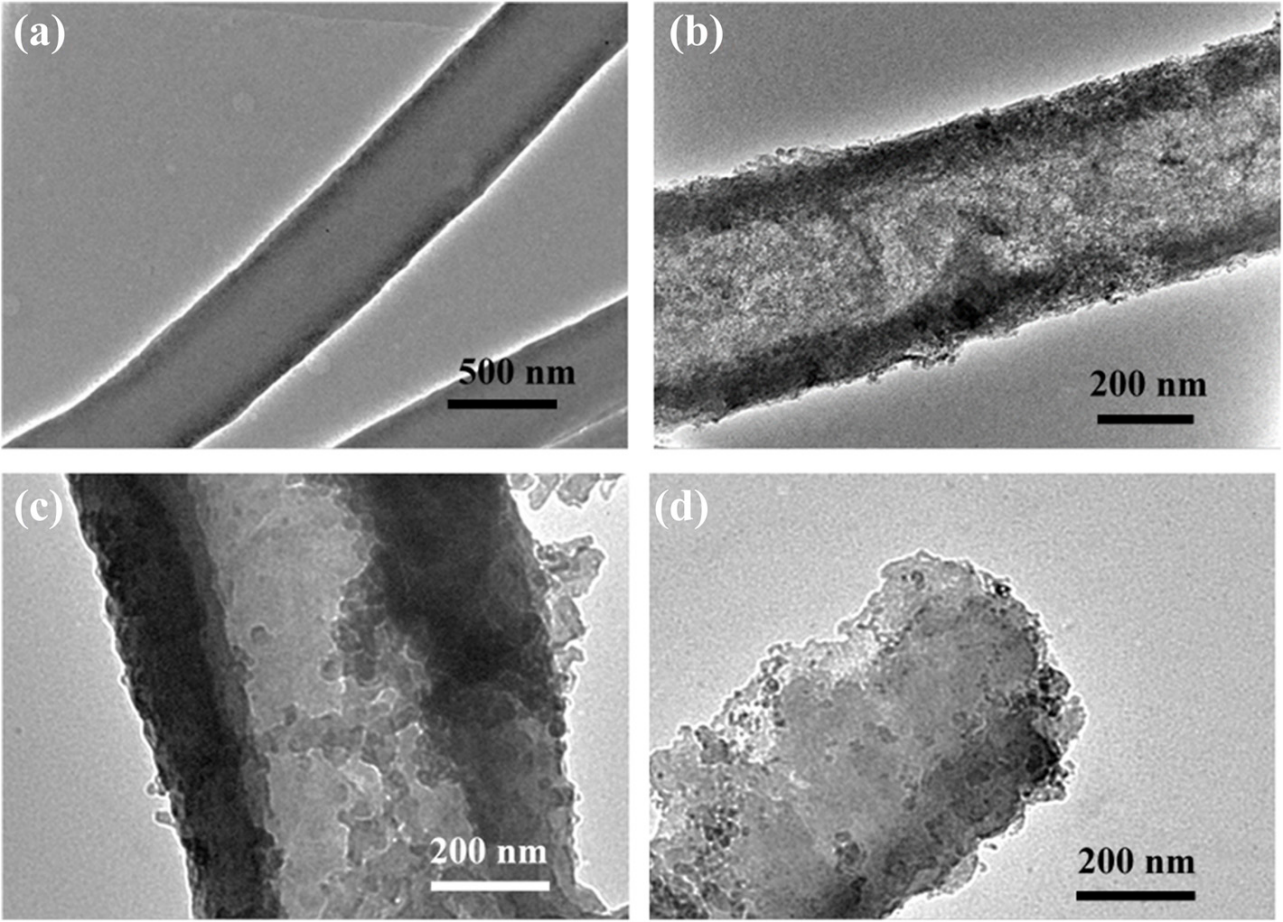
TEM images of the carbon nanofibers after carbonization. **a** CNFs from single nozzle and HCNFs from **b** normal coaxial nozzle, **c** inward coaxial nozzle, and **d** outward coaxial nozzle

The hollow morphologies were observed clearer in the TEM images as shown in Fig. 5b–d. The maintenance of core/shell structure is originated from immiscibility and thermal stability differences between PMMA and PAN during coaxial electrospinning and thermal process [15]. The formation of a hollow structure leads to the enhancement of specific surface area over the solid nanofibers (Fig. 5a). The rough surface was observed both inside and outside of the walls of all HCNF samples. This rough appearance indicates the existence of a mesoporous structure, which originated from penetration of some PMMA core to PAN shell portion during the thermal treatment [11].

As shown in Table 2, the overall diameters of the obtained HCNFs from different nozzle-end levels show no significant difference. However, the overall diameter of the HCNFs produced from the inward coaxial nozzle is slightly bigger as shown in Fig. 5b. Moreover, the shrinkage of the size diameter of the HCNFs from the inward coaxial nozzle electrospinning was only 49.30 % of their initial nanofiber diameter. This indicates that the as spun nanofibers composed of large amount of PAN shell portion and more stable in core/shell structure without core collapse morphology. In the inward coaxial system, the outer nozzle-end was set to a closer distance to the aluminium collector, which is not only pulled out the PAN solution with a high amount but also cause the PAN outer solution to form the stable compound of Taylor cones to support the inner fluid, resulting in a good stability of the core/shell structure. Meanwhile, the TEM morphology of the HCNFs produced from the outward coaxial nozzle system shows some non-uniform core/shell structure as shown in Fig. 5d. The following two factors may have partially contributed to the non-uniform core/shell structure. Initially, the PMMA core portion with lower concentration than the PAN shell portion but was pulled out with higher electrostatic force may cause the instability of jet solution during electrospinning. Thereafter, the smaller amount of the PAN solution is not enough to stabilize the compound of Taylor cones. However, this structure may have benefits for some physical properties such as an increase of the specific surface area as shown in the N_2_ adsorption measurement.

**Fig. 6:**
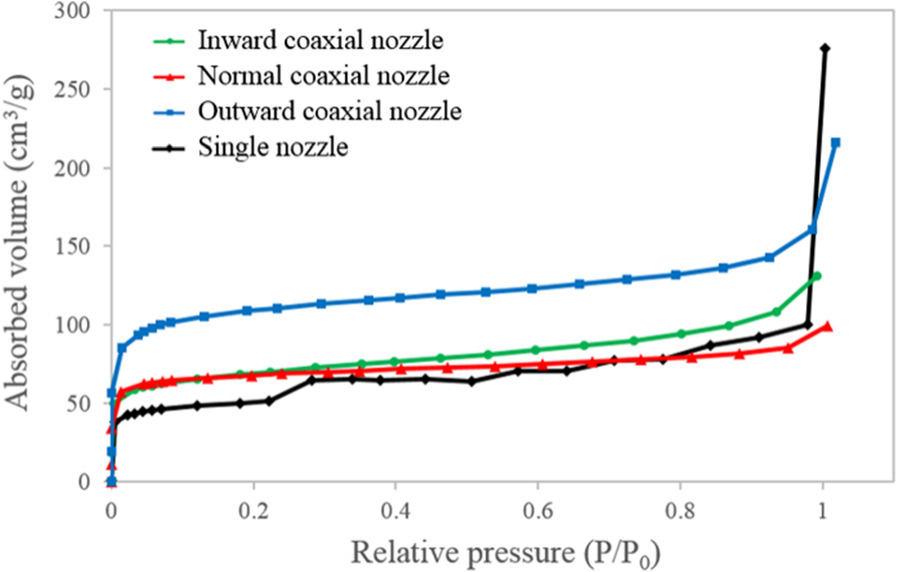
N_2_ adsorption isotherms of the HCNFs and the CNFs at −196 °C

The N_2_ adsorption isotherms (Fig. 6) show that the HCNFs produced from the inward coaxial nozzle and normal coaxial nozzle systems have a typical type-I pattern (monolayer adsorption) which indicates the presence of micropores. While the HCNFs produced from the outward coaxial nozzle and the CNFs produced from a single nozzle exhibit a type-IV isotherm (monolayer followed by multilayer adsorption), which indicates the presence of not only the micropores but also mesoporous structures [11]. From the results as shown in Fig. 6, the outward coaxial nozzle system can produce the HCNFs with highest N_2_ adsorbed volume (almost twice of the single nozzle). The BET surface area, total pore volume and average pore diameter of the HCNFs and CNFs are summarized in Table 3. From the table, the hollow morphology and porosity of both inside and outside of the walls of the HCNFs were effected to the specific surface area and pore volume of the samples. Therefore, all HCNFs samples show much larger specific surface area as compared to that of the normal CNFs. The HCNFs produced from the outward coaxial nozzle reveal the largest surface area of 278.56 m^2^ g^−1^, which is 1.69 times larger than that of the CNFs from single nozzle electrospinning. This value is also larger than those of the hollow carbon nanofibers fabricated by the same bi-component system of PMMA and PAN reported previously [13].

**Table 3:** BET surface area (*S*_BET_), total pore volume (*V*_T_), and average pore diameter of the HCNFs and CNFs

Sample	*S*_BET_ (m^2^ g^−1^)	*V*_T_ (cm^3^ g^−1^)	Avg. pore diameter (nm)
Inward coaxial nozzle	179.85	0.20	4.48
Normal coaxial nozzle	181.57	0.15	3.25
Outward coaxial nozzle	278.56	0.26	3.74
Single nozzle	164.72	0.26	6.68

**Fig. 7:**
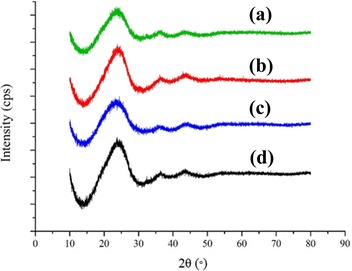
XRD patterns of the HCNFs. **a** inward coaxial nozzle, **b** normal coaxial nozzle, **c** outward coaxial nozzle, and **d** the CNFs from single nozzle

Figure 7 shows the XRD patterns of the HCNFs from the three types of coaxial nozzle and CNFs from single nozzle electrospinning. The structural features derived from XRD peaks and Raman spectrum of the samples are summarized in Table 4. From the results, the C(002) peaks were observed at 2*θ* around 24–26° indicate graphitic ordered structure of carbon. All samples show very board C(002) peak as well as large interlayer distance (c/2) than graphitic carbon (0.335 nm) and turbostratic carbon (0.344 nm) [39]. This suggested that the HCNFs and CNFs from the present work can be classified as amorphous carbon with quite low crystallinity. However, this enlarged interlayer distance of the carbon nanofibers was suitable with some applications such as anode material for sodium-ion batteries [40] since it provides high initial Coulombic efficiency, excellent rate capability, and stable cyclability.

**Table 4:** Structural features of the HCNFs and CNFs estimated from XRD and Raman measurement

Sample	C(002) peak position (°)	C(002) FWHM (°)	*I*_d_/*I*_g_	c/2 (nm)	*L*_*c*_ (nm)^a^	*L*_*a*_ (nm)^b^
Inward coaxial nozzle	25.46	7.89	0.93	0.350	1.08	4.72
Normal coaxial nozzle	23.64	6.36	0.46	0.376	1.33	9.51
Outward coaxial nozzle	23.72	9.12	0.52	0.375	0.93	8.48
Single nozzle	24.02	7.80	0.48	0.370	1.09	9.23

^a^From XRD C(002) peak and Scherrer’s equation

^b^From Raman spectra and Tuinstra’s equation

Among all samples, the HCNFs from normal coaxial nozzle show the higher C(002) peak intensity, 2.11 and 1.21 times than that of the HCNFs from inward and outward coaxial nozzle, respectively. The increase of C(002) peak intensity of the HCNFs from normal coaxial nozzle suggests the better orientation of the graphitic plane as compared to the HCNFs from inward and outward coaxial nozzle. Decrease of C(002) peak broadening (FWHM) is reflected by the decrease of *I*_*d*_*/I*_*g*_ ratio and the increase in crystallite size (both *L*_*c*_ and *L*_*a*_). Increase of the graphitization degree and crystallite size of the HCNFs from normal coaxial nozzle may arise from the smoother flow of both core and shell polymer solutions, which were pulled out from the same comparative levels of nozzle-end configuration.

The electrical conductivity of the obtained HCNFs and CNFs are shown in Table 5. Comparison with the CNFs from single nozzle electrospinning, all HCNFs samples show relatively lower electrical conductivity. Differences of the thickness among the prepared samples may have affected each of the electrical conductivities. Considering the conductance values, the HCNFs produced from the outward coaxial nozzle also show a much higher value than the CNFs produced from the single nozzle.

**Table 5:** Thickness, electrical conductance, and electrical conductivity of the HCNFs and CNFs

Sample	Thickness (μm)	Conductance (S)	Conductivity (S m^−1^)
Inward coaxial nozzle	10.82	626.00	376
Normal coaxial nozzle	13.22	882.54	648
Outward coaxial nozzle	5.58	2176.58	675
Single nozzle	10.72	1850.63	1102

Among the hollow nanofibers, the HCNFs produced from the outward and normal coaxial nozzle show higher electrical conductivity (675 and 648 S m^−1^, respectively) almost two orders of magnitude than that of the HCNFs produced from the inward coaxial nozzle system (376 S m^−1^). The high electrical conductivity of the HCNFs produced from the outward and normal coaxial nozzle may be ascribed to different reasons. For the HCNFs produced from the outward coaxial nozzle, the major contribution is its high specific surface area to volume ratio (Fig. 6 and Table 3). The hollow morphology and large amount of mesoporous and microporous structure on both inside and outside of the walls play an important role to increase the electrical conductivity [7, 8, 11]. Whereas in case of the normal coaxial nozzle, a high electrical conductivity of the HNCFs may result from a well-ordered structure with a higher graphitization degree [12, 17, 41]. The conductivity of the HNCFs and CNFs from the present work (between 370 and 1100 S m^−1^) are quite lower as compared to the previously reported results. For examples, Sebastian et al. [41] reported the conductivity of the carbon nanofibers ranged between 470 and 4100 S m^−1^. The higher electrical conductivity is mainly influenced by its higher orientation of the graphitic planes, which they suggested to be increased by an increment of synthesis temperature of the carbon nanofibers. The great effect of graphitic plane orientation also reported by Maita et al. [42] that the conductivity value of the composite nanofibers were increased almost two orders of magnitude by an increase of the degree of graphitization (*I*_g_/*I*_d_). Based on the abovementioned facts, we can imply that an increase of the electrical conductivity comes from the mixed contribution of the graphitic plane orientation and the surface area or the porosity of the HCNFs.

## Conclusions

We have clarified the influence of the applied voltage on the coaxial electrospinning of the PMMA/PAN composite nanofibers with different levels of inner nozzle-end and studied the effect of the levels of inner nozzle-end on the morphologies and physical properties of the obtained HCNFs. By using the outward coaxial nozzle system, we could produce the HCNFs with highest specific surface area of 278.56 m^2^ g^−1^, which is 1.69 times larger than that of the CNFs from single nozzle electrospinning. The XRD peaks and Raman spectrum of the HCNFs produced from normal coaxial nozzle electrospinning exhibit much higher crystallinity and graphitization degree as compared to the inward and outward coaxial nozzle systems. The high electrical conductivity of the HCNFs obtained from the outward and normal coaxial nozzle systems confirm that the electrical conductivity were affected by both of the porosity and the graphitization degree of the nanofibers.

## Abbreviations

BET: Brunauer-Emmet-Teller
c/2: interlayer spacing
CNFs: carbon nanofibers
DMF: *N*,*N*-dimethylformamide
HCNFs: hollow carbon nanofibers
*L*_*a*_: crystallite size along the c-axis
*L*_*c*_: crystallite size along the c-axis
PAN: polyacrylonitrile
PEG: polyethylene glycol
PMMA: poly(methyl methacrylate)
TEM: transmission electron microscopy
XRD: X-ray diffraction

## References

[1] Kwon W, Kim J-M, Rhee S-W (2013). Electrocatalytic carbonaceous materials for counter electrodes in dye-sensitized solar cells. J Mater Chem A.

[2] Hsu Y-H, Lai C-C, Peng Y-T, Lo C-T (2015). Preparation and characterization of interconnected carbon nanofibers. Text Res J.

[3] Kim S, Kim M, Kim YK, Hwang S-H, Lim SK (2014). Core–shell-structured carbon nanofiber-titanate nanotubes with enhanced photocatalytic activity. Appl Catal B: Environ.

[4] Mu J, Shao C, Guo Z, Zhang Z, Zhang M, Zhang P, Chen B, Liu Y (2011). High photocatalytic activity of ZnO-carbon nanofiber heteroarchitectures. ACS Appl Mater Interfaces.

[5] Yun YS, Im C, Park HH, Hwang I, Tak Y, Jin H-J (2013). Hierarchically porous carbon nanofibers containing numerous heteroatoms for supercapacitors. J Power Sources.

[6] Xu Q, Yu X, Liang Q, Bai Y, Huang Z-H, Kang F (2015). Nitrogen-doped hollow activated carbon nanofibers as high performance supercapacitor electrodes. J Electroanal Chem.

[7] Tran C, Kalra V (2013). Fabrication of porous carbon nanofibers with adjustable pore sizes as electrodes for supercapacitors. J Power Sources.

[8] Wu Y, Gao M, Li X, Liu Y, Pan H (2014). Preparation of mesohollow and microporous carbon nanofiber and its application in cathode material for lithium–sulfur batteries. J Alloys Comp.

[9] Liu B, Yu Y, Chang J, Yang X, Wu D, Yang X (2011). An enhanced stable-structure core-shell coaxial carbon nanofiber web as a direct anode material for lithium-based batteries. Electrochem Commun.

[10] Chen Y, Lu Z, Zhou L, Mai YW, Huang H (2012). Triple-coaxial electrospun amorphous carbon nanotubes with hollow graphitic carbon nanospheres for high-performance Li ion batteries. Energy Environ Sci.

[11] Park S-H, Jung H-R, Lee W-J (2013). Hollow activated carbon nanofibers prepared by electrospinning as counter electrodes for dye-sensitized solar cells. Electrochim Acta.

[12] Sebastián D, Baglio V, Girolamo M, Moliner R, Lázaro MJ, Aricò AS (2014). Carbon nanofiber-based counter electrodes for lowcost dye-sensitized solar cells. J Power Sources.

[13] El-Deen AG, Barakat NAM, Khalild KA, Kim HY (2014). Hollow carbon nanofibers as an effective electrode for brackish water desalination using the capacitive deionization process. New J Chem.

[14] Wang J, Liu Q, Gao Y, Wang Y, Guo L, Jiang G (2015). High-throughput and rapid screening of low-mass hazardous compounds in complex samples. Anal Chem.

[15] Kim C, Jeong YI, Ngoc BTN, Yang KS, Kojima M, Kim YA, Endo M, Lee JW (2007). Synthesis and characterization of porous carbon nanofibers with hollow cores through the thermal treatment of electrospun copolymeric nanofiber webs. Small.

[16] Khajavi R, Abbasipour M (2012). Electrospinning as a versatile method for fabricating coreshell, hollow and porous nanofibers. Scientia Iranica F.

[17] Wu M, Wang Q, Li K, Wu Y, Liu H (2012). Optimization of stabilization conditions for electrospun polyacrylonitrile nanofiber. Polym Degrad Stab.

[18] Deurbergue A, Oberlin A (1991). Stabilization and carbonization of PAN-based carbon fibers as related to mechanical properties. Carbon.

[19] Zussman E, Chen X, Ding W, Calabri L, Dikin DA, Quintana JP, Ruoff RS (2005). Mechanical and structural characterization of electrospun PAN-derived carbon nanofibers. Carbon.

[20] Rahaman MSA, Ismail AF, Mustafa A (2007). A review of heat treatment on polyacrylonitrile fiber. Polym Degrad Stab.

[21] Miyauchi M, Miao J, Simmons TJ, Lee J-W, Doherty TV, Dordick JS, Linhardt RJ (2010). Conductive cable fibers with insulating surface prepared by coaxial electrospinning of multiwalled nanotubes and cellulose. Biomacromolecules.

[22] Miao J, Miyauchi M, Dordick JS, Linhardt RJ (2012). Preparation and characterization of electrospun core sheath nanofibers from multi-walled carbon nanotubes and poly(vinyl pyrrolidone). J Nanosci Nanotechnol.

[23] Hong CK, Yang KS, Oh SH, Ahn J-H, Cho B-H, Nah C (2008). Effect of blend composition on the morphology development of electrospunfibres based on PAN/PMMA blends. Polym Int.

[24] Li L, Jiang Z, Li M, Li R, Fang T (2014). Hierarchically structured PMMA fibers fabricated by electrospinning. RSC Adv.

[25] Zander NE, Strawhecker KE, Orlicki JA, Rawlett AM, Beebe TP (2011). Coaxial electrospun poly(methyl methacrylate)-polyacrylonitrile nanofibers: atomic force microscopy and compositional characterization. J Phys Chem B.

[26] Minella AB, Pohl D, Täschner C, Erni R, Ummethala R, Rümmeli MH, Schultz L, Rellinghaus B (2014). Silicon carbide embedded in carbon nanofibres: structure and band gap determination. Phys Chem Chem Phys.

[27] Wang Y, Serrano S, S-Aviles JJ (2002). Conductivity measurement of electrospun PAN-based carbon nanofiber. J Mater Sci Letters.

[28] Bedi JS, Lester DW, Fang YX, Turner JFC, Zhou J, Alfadul SM, Perry C, Chen Q (2013). Electrospinning of poly(methyl methacrylate) nanofibers in a pump-free process. J Polym Eng.

[29] Zhang C, Yuan X, Wu L, Han Y, Sheng J (2005). Study on morphology of electrospun poly(vinyl alcohol) mats. Eur Polym J.

[30] Reneker DH, Chun I (1996). Nanometre diameter fibres of polymer, produced by electrospinning. Nanotechnol.

[31] Yuan X, Zhang Y, Dong C, Sheng J (2004). Morphology of ultrafine polysulfone fibers prepared by electrospinning. Polym Int.

[32] Jalili R, Morshed M, Ravandi SAH (2006). Fundamental parameters affecting electrospinning of PAN nanofibers as uniaxially aligned fibers. J Appl Polym Sci.

[33] Deitzel JM, Kleinmeyer J, Harris D, Beck TNC (2001). The effect of processing variables on the morphology of electrospun nanofibers and textiles. Polymer.

[34] Zuo W, Zhu M, Yang W, Yu H, Chen Y, Zhang Y (2005). Experimental study on relationship between jet instability and formation of beaded fibers during electrospinning. Polym Eng Sci.

[35] Enz E, Baumeister U, Lagerwall J (2009). Coaxial electrospinning of liquid crystal-containing poly(vinylpyrrolidone) microfibers. J Org Chem.

[36] Li D, Xia Y (2004). Direct fabrication of composite and ceramic hollow nanofibers by electrospinning. Nano Lett.

[37] Pillay V, Dott C, Choonara YE, Tyagi C, Tomar L, Kumar P, du Toit LC, Ndesendo VMK (2013) A review of the effect of processing variables on the fabrication of electrospun nanofibers for drug delivery applications. J Nanomater:1–22. doi:10.1155/2013/789289

[38] Tuinstra F, Koenig JL (1970). Raman spectrum of graphite. J Chem Phys.

[39] Vogel W, Hosemann R (1979). The paracrstalline nature of pyrolytic carbons. Carbon.

[40] Qie L, Chen W, Xiong X, Hu C, Zou F, Hu P, Huang Y (2015). Sulfur-doped carbon with enlarged interlayer distance as a high-performance anode material for sodium-ion batteries. Adv Sci.

[41] Sebastian D, Ruiz AG, Suelves I, Moliner R, Lazaro MJ (2013). On the importance of the structure in the electrical conductivity of fishbone carbon nanofibers. J Mater Sci.

[42] Maitra T, Sharma S, Srivastava A, Cho Y-K, Madou M, Sharma A (2012). Improved graphitization and electrical conductivity of suspended carbon nanofibers derived from carbon nanotube/polyacrylonitrile composites by directed electrospinning. Carbon.

